# Factors Influencing Internal Medicine Resident Beta-Blocker Discontinuation in Acute Decompensated Heart Failure

**DOI:** 10.1016/j.cjco.2025.07.013

**Published:** 2025-07-30

**Authors:** Daniel Boctor, Samuel B. Brusca, Gurpreet Dhaliwal, Matthew D. Ponzini, Noelle Boctor, Connor G. O’Brien

**Affiliations:** a Department of Medicine, University of California, San Francisco, San Francisco, California, United States; b Division of Cardiology, Department of Medicine, University of California San Francisco, San Francisco, California, United States; c Medical Service, Veterans Affairs, San Francisco Health Care System, San Francisco, California, United States; d Clinical and Translational Science Center, University of California, Davis, Sacramento, California, United States; e Department of Internal Medicine, University of California, Davis, Sacramento, California, United States

**Keywords:** acute decompensated heart failure, beta-blocker withdrawal, heart failure, mechanistic reasoning, medical decision-making, resident physicians

## Abstract

**Background:**

Heart failure is one of the leading causes of hospital admissions in North America. Although guidelines support the continuation of beta blockers on admission, hemodynamic considerations and mechanistic reasoning may prompt beta blocker discontinuation even in the absence of contraindications. Resident physicians often face this dilemma and are an important group in which to evaluate this decision-making.

**Methods:**

Internal medicine residents at two institutions were presented with two scenarios: 1) whether to continue outpatient metoprolol succinate for a patient without evidence of shock admitted with acute decompensated heart failure (ADHF) and 2) beta blocker selection during a patient’s index presentation with heart failure.

**Results:**

142 of 287 (49.5%) residents responded to the survey. In scenario 1, 61% of residents discontinued metoprolol succinate on admission. The top three concerns about continuing metoprolol were precipitating cardiogenic shock, discomfort with the vital signs range, and attending physician disagreement. In scenario 2, 74% of participants initiated metoprolol succinate, 25% chose carvedilol, and only 1 participant chose bisoprolol.

**Conclusions:**

Drivers of inpatient beta blocker discontinuation should be considered by internal medicine training programs and heart failure guideline writers when opportunities arise to enact practice changes that align with evidence.

## Résumé

**Contexte:**

L’insuffisance cardiaque est l’une des principales causes d’hospitalisation en Amérique du Nord. Bien que les lignes directrices appuient la poursuite du traitement par des bêtabloquants lors d’une hospitalisation, un raisonnement fondé sur les considérations hémodynamiques et mécanistiques pourrait prôner l’arrêt du traitement par des bêtabloquants même en l’absence de contre-indications. Les médecins résidents, qui font souvent face à ce dilemme, constituent un important groupe au sein duquel nous avons évalué la prise de cette décision.

**Méthodologie:**

On a présenté deux scénarios à des résidents en médecine interne de deux établissements : 1) poursuivre le traitement par le succinate de métoprolol amorcé en externe chez un patient hospitalisé en raison de la décompensation d’une insuffi-sance cardiaque aigüe sans signes probants de choc cardiogénique; et 2) choisir un bêtabloquant pour un patient dont le motif d’admission de référence était une insuffisance cardiaque.

**Résultats:**

En tout, 142 résidents sur 287 (49,5 %) ont répondu au sondage. Pour ce qui est du premier scénario, 61 % des résidents ont mis fin au traitement par le succinate de métoprolol lors de l’admission du patient. Les trois principales préoccupations à l’égard de la poursuite du traitement par le métoprolol étaient le risque de déclenchement d’un choc cardiogénique, une hésitation liée aux mesures des signes vitaux et le désaccord du médecin traitant. Quant au deuxième scénario, 74 % des médecins participants ont amorcé un traitement par le succinate de métoprolol, 25 % ont prescrit le carvédilol et 1 seul participant a opté pour le bisoprolol.

**Conclusions:**

Les motifs d’abandon du traitement par des bêtabloquants lors de l’hospitalisation devraient être couverts par les programmes de formation en médecine interne et pris en compte par les auteurs des lignes directrices sur l’insuffisance cardiaque, de façon à mettre en œuvre un changement de pratique correspondant aux données probantes lorsqu’une telle occasion se présente.

Medical decision-making is shaped by evidence, guidelines, pathophysiologic principles, and experience. These tenets often come into conflict in clinical scenarios where the physician invokes a mechanistic principle that is not aligned with evidence-based practice. Although evidence-based medicine has always supported the incorporation of patient-specific factors when making therapeutic decisions, overreliance on mechanistic reasoning pertaining to a specific patient can impede the implementation of salutary treatments. One practice that captures the tension between evidence-based practice and mechanistic reasoning is the discontinuation of beta-blockers (BBs) in patients with acute decompensated heart failure (ADHF). ADHF is defined by the **A**merican **H**eart **A**ssociation/**A**merican **C**ollege of **C**ardiology/**H**eart **F**ailure **S**ociety of **A**merica (AHA/ACC/HFSA) as a complex syndrome with signs and symptoms of congestion that result from structural or functional cardiac abnormalities associated with evidence of elevated natriuretic peptides.^1^

Resident physicians are an important group in which to explore medical decision-making, as they are frequently the clinicians who decide whether to discontinue BBs at the time of admission. In addition, the behaviors of residents persist long after residency and, as future attending physicians, they represent the internal medicine workforce that will continue practice norms and drive future medical education.^2^^,^^3^ Finally, exploring how residents interpret and apply clinical guidelines can be a valuable source of information for future guideline formulation.

Before the 1980s, BBs were strictly contraindicated in patients with heart failure based on the rationale that a weak cardiac reserve needed adrenergic support to prevent circulatory decompensation (the hemodynamic model of heart failure).^4^ Numerous landmark trials, building on the shift to the neurohormonal model of heart failure, subsequently established BBs (specifically bisoprolol, carvedilol, and metoprolol succinate) as a pillar of guideline-directed medical therapy (GDMT) with mortality benefits.^5^, ^6^, ^7^ Nevertheless, recent data have shown that more than a third of eligible heart failure patients are not prescribed a BB, and those who are frequently on lower-than-recommended target dosages.^8^

Despite the importance of inpatient GDMT (including BBs) prescription to outcomes and overall GDMT compliance, few studies have specifically examined this population. In the **B**eta**-**Blocker **Con**tinuation **V**s. **In**terruption in Patients With **C**ongestive Heart Failure Hospitaliz**ed** (B-CONVINCED) trial, continuation of outpatient BB treatment in patients admitted with ADHF and left ventricular ejection fraction (LVEF) < 40% did not worsen outcomes compared with discontinuing the BB.^9^ Participants in the continuation arm had a higher likelihood of being on their BB at 3 months postdischarge. This is consistent with other studies that linked BB discontinuation during ADHF admission with increased mortality and higher rates of rehospitalization.^10^, ^11^, ^12^, ^13^ The 2022 AHA/ACC/HFSA Guideline for the Management of Heart Failure recommends maintaining BB treatment in patients without evidence of shock who are admitted with ADHF.^1^

Despite these findings and guideline recommendations, BBs are discontinued in 22%*-*73% of all heart failure admissions.^10^^,^^13^ Some authors have speculated that the practice of discontinuing BB on admission arises from the concern of the negative inotropy precipitating cardiogenic shock.^9^^,^^14^ However, no studies have investigated the reasoning driving this practice in patients without evidence of shock.^13^

We conducted a multicenter study to explore resident physicians’ decision-making surrounding BB prescription on admission for ADHF. To identify the effects of mechanistic reasoning on decision-making, we also asked participants about the initiation of BB for GDMT prior to discharge.

## Methods

### Participants

Eligible participants were all internal medicine residents (categorical, primary care, and combined internal medicine/psychiatry programs) in 2 academic programs in California, United States. Preliminary residents rotating in internal medicine were excluded. A total of 287 eligible residents participated (186 from the University of California, San Francisco [UCSF] and 101 from the University of California, Davis [UCD]). All participants provided informed consent.

### Survey design

We designed a survey based on the theory of planned behavior, a model used to evaluate human decision-making.^15^^,^^16^ This theory states that attitude (knowledge and beliefs), subjective norms (perceptions and practices of peers), and perceived behavioral control (belief about one’s capabilities) collectively influence decision-making.

### Survey content

Participants were given 2 clinical cases. The first case was a patient on metoprolol succinate 50 mg/day admitted for ADHF without evidence of shock (Supplemental Appendix S1). The patient’s blood pressure was 98/59 mm Hg (baseline 118/65 mm Hg), heart rate 59 beats/min, and oxygen saturation 95% on 4-L nasal cannulation, which was a new oxygen requirement. This heart rate value was chosen because it is above the definition of 50 beats/min for bradycardia used in the 2018 ACC/AHA/Heart Rhythm Society Guideline on the Evaluation and Management of Patients With Bradycardia and Cardiac Conduction Delay, yet it is below the traditional threshold of 60 beats/min that many clinicians define as normal.^17^ The 2022 AHA/ACC/HFSA Guideline states that “true contraindications [for beta-blocker continuation] are rare, such as advanced degree atrioventricular block for beta blockers in the absence of pacemakers, [or] cardiogenic shock that may preclude use of certain medications until resolution of shock state.”^1^ None of these contraindications were present in the study scenario given to participants.

The participants were asked if, in the absence of contraindications (bradycardia, severe hypotension, signs of shock), they would continue this patient’s BB, hold it until discharge, or hold it past discharge. If they indicated either option corresponding to holding the BB, they were given a menu of 9 concerns to explain their choice. The menu of concerns was designed using the TPB, with each option assessing attitude, subjective norms, or perceived behavioral control relating to BB prescription. There was an option to describe any other concern not presented. Participants were invited to rank each concern from 1 to 9, with 1 being the highest concern. For their top concern, the participants were asked to elaborate on their reasoning in a free text response. Respondents were also asked how often they hold BBs for ADHF in their practice and if they believe that BBs cause cardiogenic shock (both in the absence of contraindications).

In the second case, participants were given the scenario of a patient with a new diagnosis of heart failure with reduced ejection fraction admitted for ADHF who was decongested to euvolemia and ready for initiation of GDMT. This scenario evaluated which BB the resident would prescribe: metoprolol succinate, carvedilol, or bisoprolol. Based on their choice, they were asked to rank a list of possible reasons, each aimed at exploring a component of the TPB. This scenario was developed to determine whether mechanistic reasoning influences residents in a situation of clinical equipoise given the limited comparative data between carvedilol, metoprolol succinate, and bisoprolol in heart failure.^18^^,^^19^

### Survey administration

Residents at both institutions received an e-mail invitation to complete an online Qualtrics survey. The survey collection window was 4 weeks. Electronic reminders were sent weekly. Residents who completed the survey had the option to enroll in a drawing for a $25 gift card. Participation was voluntary and anonymous. If the participants elected to enroll in the drawing, they were asked to enter their e-mail in a separate link that was not tied to their survey responses. Our study was approved separately by the institutional review boards of the UCSF and UCD.

### Data analysis

Respondent demographics were obtained including age, gender, residency program, postgraduate year, and state in which they attended medical school. The medical school states were then combined into the **G**roup on **S**tudent **A**ffairs (GSA) regions of the **A**ssociation of **A**merican **M**edical **C**olleges (AAMC): central, southern, northeastern, and western regions of the United States.

We tabulated the frequency count of the ranked concerns in scenario 1 to analyze how often each of the 9 concerns appeared as a top 3 concern for the participants. We calculated a mean rank of each concern to establish their relative importance. For both scenarios, categorical variables were summarized as frequency percentages. Group comparisons were made using the χ^2^ test or Fisher exact test, as appropriate. All analyses were conducted using R version 4.4.1 (R Foundation for Statistical Computing, Vienna, Austria). For scenario 2, we tabulated a frequency count of the choice of BB to quantify how often each medication was chosen. Finally, we performed a qualitative analysis of the free-text responses in both scenarios to identify recurrent themes. Three authors each conducted separate qualitative content analyses to identify themes (inductive coding) in the data and then met to reconcile coding differences.^20^

## Results

**Figure 1:**
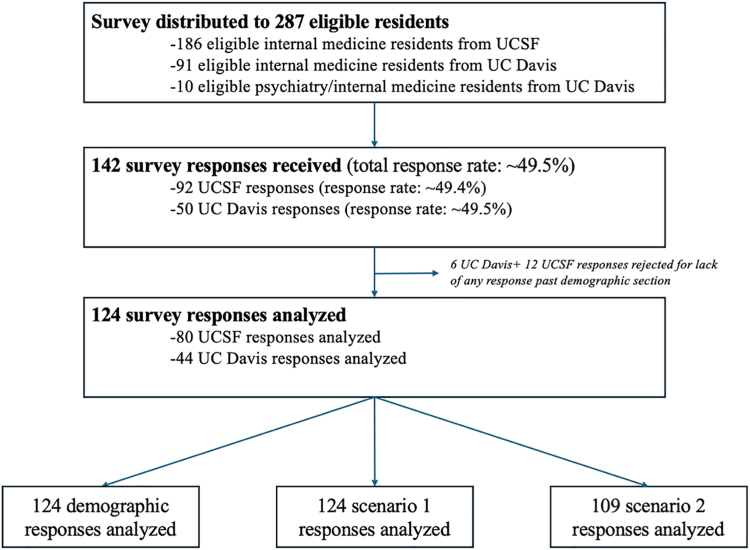
Flowchart of participant survey responses. Response rate, number of rejected responses, and number of analyzed responses are presented, by institution. UCSF, University of California, San Francisco.

A total of 142 of 287 residents responded to the survey (response rate 49.5%). Ninety-two participants were from UCSF (response rate 49.5%) and 50 participants were from UCD (response rate 49.5%). Most (76.8%) participants completed the entire survey. Partial responses with only demographic information and no responses to any scenario questions were excluded from the analysis. This eliminated 6 responses from UCD and 12 responses from UCSF, leaving 124 responses in the final analysis (Fig. 1). Most respondents were women (61.3%) and between 29 and 32 years of age (63%). Nearly half of the participants (49%) were trained in a medical school in the western United States (Table 1 and Supplemental Table S1).

**Table 1:** Demographic characteristics of survey respondents included in analysis with metoprolol prescription decision on admission

Characteristic	Total number of respondents, n = 124	Continue metoprolol on admission, n = 48	Hold metoprolol on admission, n = 76	*P* value
Year in training				0.0882∗
Postgraduate year 1	35 (28%)	9 (19%)	26 (34%)	
Postgraduate year 2	50 (40%)	24 (50%)	26 (34%)	
Postgraduate year 3	38 (31%)	14 (29%)	24 (32%)	
Postgraduate year 4	1 (0.8%)	1 (2.1%)	0 (0%)	
Age, years				0.23†
> 32	13 (10%)	2 (4.2%)	11 (14%)	
25-28	33 (27%)	15 (31%)	18 (24%)	
29-32	78 (63%)	31 (65%)	47 (62%)	
Sex				0.43†
Female	76 (61%)	27 (56%)	49 (64%)	
Male	48 (39%)	21 (44%)	27 (36%)	
Current institution				0.33†
UCSF	80 (65%)	28 (58%)	52 (68%)	
UCD	44 (35%)	20 (42%)	24 (32%)	
Geographic region of medical school attended in United States				0.63†
Central	18 (15%)	7 (15%)	11 (14%)	
Northeastern	28 (23%)	8 (17%)	20 (26%)	
Southern	17 (14%)	8 (17%)	9 (12%)	
Western	61 (49%)	25 (52%)	36 (47%)	

Data expressed as number of respondents (%).

UCD, University of California, Davis; UCSF, University of California, San Francisco.

∗ Fisher exact test.

† Pearson χ^2^ test.

### Scenario 1: Statistical analysis

In the first scenario, residents were presented with the case of a patient admitted for ADHF (with no contraindications to BBs) and asked if they would continue the patient’s outpatient metoprolol prescription. Most residents (76 of 124, 61%) indicated that they would hold metoprolol on admission and restart it before discharge, with fewer (48 of 124, 39%) indicating that they would continue the medication on admission. No residents stated that they would hold metoprolol on admission and wait to restart it as an outpatient (Table 1).

**Figure 2:**
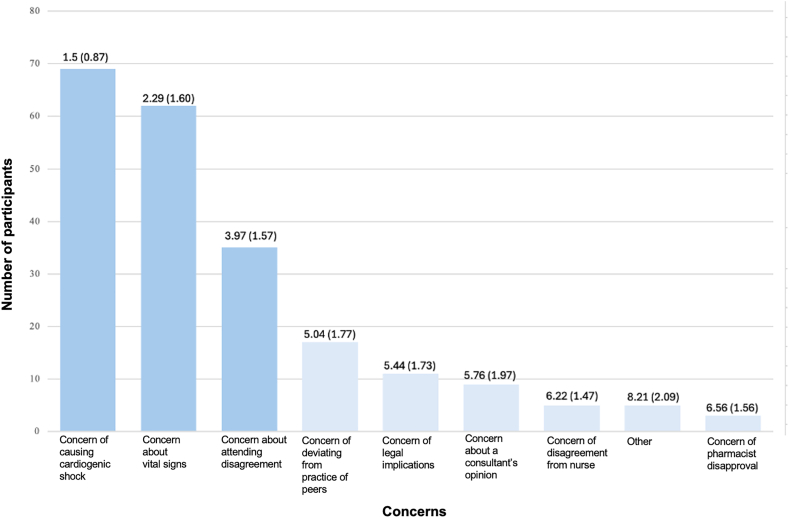
Ranking of concerns driving the decision to discontinue metoprolol on admission in scenario 1. The first number at the top of each bar represents the mean rank of each concern (with the standard deviation in parentheses).

Participants who chose to hold the BB on admission were asked to rank their top 3 concerns compelling them to hold the medication. Ninety-six percent stated concern for cardiogenic shock (mean rank 1.5 of 9), 86% noted discomfort with the patient’s vital signs (mean rank 2.29 of 9), and 49% mentioned anticipated attending disagreement (mean rank 2.97 of 9). Seventeen percent of residents also indicated the concern of deviating from the practice of their peers (Fig. 2).

Reflecting on their routine practice, only 13% (15 of 115) of residents responded they never hold BBs in the absence of contraindications. Forty-two percent (48 of 115) of residents stated they hold BBs on admission in the absence of contraindications in at least a quarter of their ADHF cases. When participants were asked if BBs can cause cardiogenic shock in the absence of the prespecified contraindications, nearly half (54 of 115, 47%) were unsure and approximately a quarter (30 of 115, 26%) answered yes (Table 2).

**Table 2:** Participants’ decisions and beliefs around prescribing metoprolol in ADHF

In patients with acute heart failure exacerbation, how often do you hold outpatient metoprolol on admission in the absence of new bradycardia, severe hypotension, or signs of shock?	n (%)
Never (0%)	15 (13.0%)
Occasionally (1%-24%)	52 (45.2%)
Often (25%-49%)	24 (20.9%)
Routinely (50%-99%)	22 (19.1%)
Always (100%)	2 (1.7%)
No response	9
In patients with acute heart failure exacerbation, can metoprolol cause cardiogenic shock in the absence of new bradycardia, severe hypotension, or signs of shock?	
No	31 (27.0%)
Unsure	54 (47.0%)
Yes	30 (26.1%)
No response	9

ADHF, acute decompensated heart failure.

**Figure 3:**
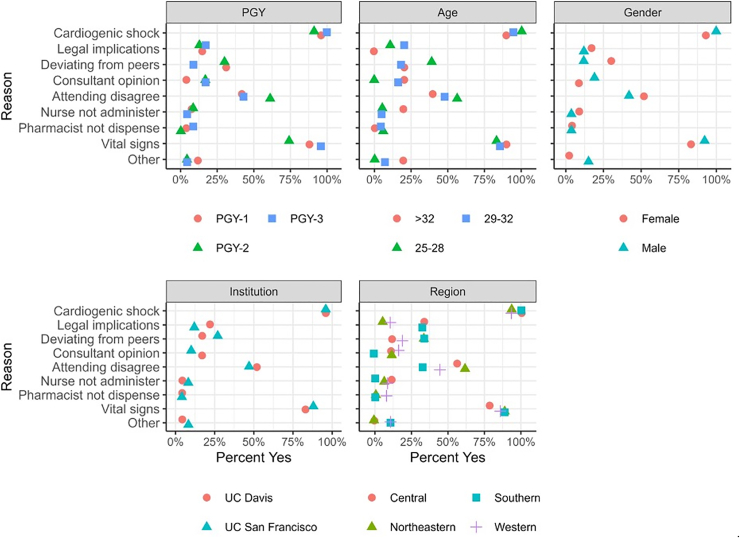
Scatterplot of concerns driving decision to discontinue metoprolol on admission by demographic parameters. The Y axis lists the concerns driving the decision to discontinue metoprolol, and the X axis is the percentage of respondents for whom the concern appeared in the top 3. Different icons are used to convey the different demographic groups. PGY, postgraduate year; UC, University of California.

There was no statistical difference in answer choices when residents were analyzed by institution, regional location of medical school, year of training, or sex (Table 1 and Fig. 3).

### Scenario 1: Qualitative analysis

Thematic analysis of comments from free-text responses elaborating on participants’ top concern about metoprolol succinate continuation highlighted 3 psychological drivers of discontinuation: 1) risk of acute clinical deterioration, 2) deference to attending physicians and protocols, and 3) availability of an alternative BB strategy (eg, switching from metoprolol succinate to tartrate on admission) (Table 3).

**Table 3:** Participants’ responses when asked to elaborate on their motivation to withhold long-acting metoprolol upon admission for ADHF

Theme	Subtheme	Representative quote
Risk of acute clinical deterioration	Concern about the vital signs	“Since [heart rate] is already low at 59 and has a low BP, would be concerned that could worsen with the metoprolol.”
	Concern about precipitating shock	"[systolic blood pressure] is almost 20 points lower than her baseline, would be worried about impending cardiogenic shock."
	Concern about ICU exposure	"Commit this person to the consequences of being in the ICU such as invasive lines, longer hospital stay, ICU delirium."
	Concern about precipitating respiratory distress	“Worsening congestion causing progressive pulmonary edema/respiratory distress.”
Deference to attending/protocol	Deference to attending/protocol	[Concern about] "scolding from cardiology": "We have been taught not to prescribe beta-blockers during the acute phase of a [heart failure exacerbation]."
Availability of an alternative BB strategy	Switch to short-acting BB while in ADHF	"I don't see the downside of switching them to short acting [metoprolol], to be able to more easily respond to clinical changes."
	Delay resumption until outpatient adherence is confirmed	"Would want to clarify adherence before continuing BB” [reasonable to continue if taking reliably and no evidence of shock, but would not do new start].

ADHF, acute decompensated heart failure; BB, beta-blocker; BP, blood pressure; HR, heart rate; ICU, intensive care unit.

### Scenario 2: Statistical analysis

In the second scenario, residents were presented with the case of a patient admitted for ADHF who was successfully decongested to euvolemia, and then asked which evidence-based BB they would initiate at discharge. The majority (81 of 109, 74.3%) chose metoprolol succinate, whereas the others chose carvedilol (27 of 109, 24.7%). Only 1 participant chose bisoprolol (Supplemental Tables S2 and S3).

### Scenario 2: Qualitative analysis

Participants who chose metoprolol succinate indicated that the 2 principal reasons for their decision were that metoprolol succinate is administered once daily (55 of 81, 68%) and that it is what most of their peers prescribe (27 of 81, 33%). Among the 19 participants who also provided write-in responses, nearly all felt that metoprolol would cause less reduction in blood pressure than carvedilol and therefore allow initiation of other GDMT agents. The participants who chose carvedilol indicated that the principal reason for their decision was evidence that carvedilol had greater efficacy (eg, blood pressure effect) (15 of 27, 55%). A similar number of participants (9 of 27, 33%) indicated that afterload reduction and attending physician preferences drove them to choose carvedilol.

## Discussion

### Residents’ attitudes toward BBs

In this study we have explored the factors that drive internal medicine residents to discontinue outpatient BBs when admitting a patient with ADHF in the absence of BB contraindications (Central Illustration). This scenario was examined because it represents a commonly faced clinical decision both on cardiology and medicine rotations and because clinical practice often differs from that recommended in the guidelines.

**Central Illustration:**
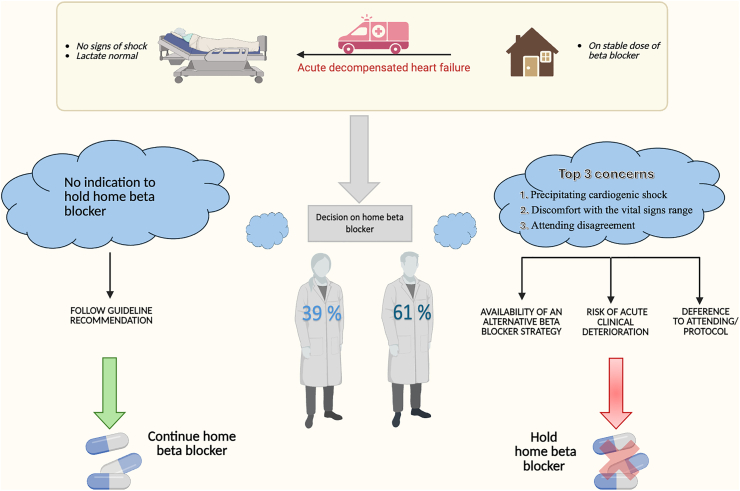
In this study, 61% of resident physicians held beta-blockers on admission in patients with acute decompensated heart failure without any signs of shock. The top 3 concerns driving this decision are listed below.

Resident attitudes (the first dimension of the TPB, characterized as knowledge and beliefs) toward BB in this scenario were varied. Nearly half (47%) were uncertain whether BBs can cause shock in the absence of contraindications and over a quarter (26%) stated that BBs can cause shock in the absence of contraindications. Many residents foresaw potential negative consequences of continuing BBs, including cardiogenic shock, bradycardia, and transfer to the intensive care unit.

### The role of mechanistic reasoning

These concerns highlight the tension between mechanistic reasoning and evidence-based medicine.^21^ Historically, BBs were contraindicated in heart failure due to the concern of negative inotropy precipitating cardiogenic shock. This mechanistic reasoning posited that pharmacologic antagonism of the adrenergic response is deleterious for a heart with diminished contractility. Mechanistic reasoning---which makes inferences from mechanisms (cause-and-effect relationships between different parts of the body or its systems, often established through physiology, pharmacology, or molecular biology) to advance claims that an intervention will produce a patient-relevant outcome---may be necessary when no or low-quality evidence exists to guide a decision.^21^ However, when high-quality evidence (often captured as level 1 recommendations) to guide a decision exists, overriding it with an inferential chain linking an intervention (BB) to an outcome (hemodynamic changes) may be counterproductive.

Two of the 3 themes describing the motivation to withhold BBs in this study created such an inferential link but differed in their time horizon and focus. The theme of “risk of acute deterioration” conveyed a decision to hold BB without a predefined plan to restart it, suggesting a concern about clinical decompensation. In contrast, the “availability of an alternative BB strategy” theme conveyed a conditional strategy that could be employed in lieu of automatic continuation of outpatient BBs. This strategy foresaw how beta blockade could be reinitiated when certain criteria were met, such as tolerance of a short-acting beta-blockade trial or confirmation of outpatient adherence first.

One study looking at drivers of metformin discontinuation on admission, characterized as a contestable standard of care (a practice that persists widely despite lack of benefit or evidence of harm), revealed heightened concerns about lactic acidosis (a knowledge issue), attending physician disagreement (a hierarchical influence), and peer perception (a social effect).^16^ In this current study, metoprolol succinate continuation demonstrated parallel concerns: worsening clinical status (knowledge) and deference to attending/protocol (hierarchy). The decision to discontinue metformin was also facilitated by a readily available alternative approach (inpatient sliding scale insulin). The current study has similarly revealed alternative BB strategies that gave the clinicians options other than their less-favored path of long-acting BB continuation.

Scenario 2 (deciding which BB to use in GDMT) also highlighted mechanistic reasoning in decision-making, but in a scenario where clinical equipoise exists.^18^^,^^19^ When asked to elaborate on their reasoning, mechanistic pharmacology (pharmacodynamics) was frequently referenced, such as the dosing schedule of metoprolol succinate, the participants’ beliefs about differential blood pressure effects between metoprolol and carvedilol, and the predicted afterload effects of the latter. Social factors, including the opinions of attending physicians and the practices of their peers, were also drivers of BB choice. This highlights the effect of the second dimension of the TPB (subjective norms) on residents’ decision-making. The influence of the third dimension of the TPB (perceived behavioral control) was seen in both scenarios---for example, deferral to protocols for BB discontinuation on admission (scenario 1) or following attending physician preferences regarding discontinuation (scenario 1) or prescription (scenario 2).

We found no statistical difference in decision-making based on current institution, region of medical school attendance, postgraduate year, or sex. There was also no statistical difference between institutions on the top 3 concerns chosen to justify holding metoprolol on admission. This suggests that the instinct to invoke or consider mechanistic reasoning develops early in medical training. The data presented in this work highlight the origins of clinical decisions with potentially clinically deleterious implications.

### The heterogeneous definition of cardiogenic shock

The 2022 AHA/ACC/HFSA Guideline for the Management of Heart Failure states that BBs should be held in cases of cardiogenic shock, but should be continued in cases of “mild or transient reductions in blood pressure.”^1^ However, the definition of cardiogenic shock is heterogeneous and has potentially become more ambiguous with the increased use of the **S**ociety for **C**ardiovascular **A**ngiography and **I**ntervention (SCAI) cardiogenic shock stages, with multiple variations promoted by shock research groups.^22^^,^^23^ In our survey, the patient in scenario 1 with stable ADHF would be classified as having SCAI shock stage A, which is defined as patients “at risk” for cardiogenic shock.^22^ Such patients appear clinically stable, with evidence of congestion but no hypoperfusion or hemodynamic instability, essentially identifying all hospitalized patients with classic “warm and wet” ADHF. In these cases, BBs should typically be continued, and few clinicians are likely inclined to classify them as being in shock at all. The inclusion of these at-risk patients in shock staging systems could therefore be confusing and counterproductive for clinicians, potentially leading to inappropriate GDMT cessation.

For SCAI stage B, in which patients are in the beginning phases of shock, BB prescription may be more complex. At face value, as these patients are being defined as “in shock,” BB should likely be discontinued. However, there are nuances to how SCAI and other groups, such as the **C**ardiogenic **S**hock **W**orking **Group** (CSWG), define SCAI stage B, with the former defining it as hypotension without hypoperfusion and the CSWG defining it as either hypotension or hypoperfusion. One can construct scenarios, such as a mildly hypotensive patient with a normal serum lactate or a hypertensive patient with a mildly elevated serum lactate, where BB administration may still be reasonable.

Although the SCAI shock staging has improved our ability to risk-stratify and prognosticate cardiogenic shock, the broad definition of stages A and B may lead to more patients being diagnosed with cardiogenic shock on admission. Greater diagnosis of shock may lead to mechanistic reasoning that interferes with clinicians providing evidence-based standard care for ADHF patients presenting in lower SCAI shock stages, namely continuing BBs when no contraindications exist. Given the variability in the definitions of SCAI shock staging, the authors suggest that SCAI staging should be used in conjunction with hemodynamic variables, measures of perfusion, serology, and the patient’s clinical picture to decide on BB prescription. Future SCAI consensus statements could offer guidance on BB decision-making in SCAI cardiogenic shock stages A and B. Furthermore, modeling the appropriate continuation of BBs in study question banks and certification exams is also necessary as this represents an important tool in influencing knowledge consolidation for trainees.

### Study limitations

Our study has several limitations. First, the survey had a modest response rate. Second, clinical decision-making in a survey format is inherently different than the nuanced and complex situations confronted in clinical practice. Qualitative analyses with formal interviews or simulation could uncover a broader characterization of factors that inhibit the continuation of BBs. Although attending physicians were cited as a factor in decision-making (both as purveyors of knowledge and with hierarchical control on decisions), we were unable to determine whether medicine or cardiology attending physicians (both of whom supervise ADHF admissions) were being referenced in responses. Ambiguity pertaining to the terms used in the survey or responses may lead participants and investigators to lack a shared mental model of the concepts in question (eg, the aforementioned confusion regarding “cardiogenic shock”). Furthermore, we must acknowledge that residents may make clinical decisions to align with their attending physician’s (who are evaluating them) prescription habits, even with the knowledge that they go against guideline-directed care. In this way, it is difficult to ascertain if a resident’s preferred clinical decision is being actively suppressed or if they would make a different decision once in independent practice. Finally, many of the studies of BBs in ADHF are 10-20 years old, which could limit the authority of current guidelines. Given the focus on newer therapeutics, however, it is unlikely that large studies will be conducted to provide new data on this issue.

We chose the TPB as our behavior framework because it incorporates internal (attitudes) and external (subjective norms and perceived behavioral control) factors as predictors of action. An important omission in the theory of planned behavior is the failure to assess emotions in decision-making.^24^ Other frameworks of motivation, including Goal Setting Theory^25^ and the **C**apability, **O**pportunity, **M**otivation, **B**ehavior (COM-B) model,^26^ could have revealed different dimensions of the reasoning process.

## Conclusions

To our knowledge, this is the first study to examine the cognitive factors that drive internal medicine residents’ decision to continue or withhold outpatient BB treatment upon admission for ADHF. The decision to discontinue BBs was primarily driven by the risk of hemodynamic decompensation, highlighting the cognitive tension that arises between mechanistic reasoning and guideline-concordant care. The latter has the best chance of influencing frontline clinician management where there is coherence between guideline society statements and a shared understanding of common terms (like “shock”). Our study exposes a potential conflict in the literature between heart failure guidelines (which promote continued BB prescription in the absence of shock) and SCAI shock staging, which defines all admitted ADHF patients as at least SCAI cardiogenic shock stage A.

Future studies should examine practice patterns in clinicians at different career stages, including advanced practice providers, subspeciality fellows, and attending physicians, who are all governed by their own attitudes, subjective norms, and perceived behavioral control. Specifically, a study assessing the practice of faculty supervising residents would help determine whether resident education is deficient or erroneous. Change led by clinical leaders will influence the decision-making of residents who will one day become attending physicians and will model the practices they learned in training.
