# Supplementary material for: Factors Influencing Internal Medicine Resident Beta-Blocker Discontinuation in Acute Decompensated Heart Failure
Source: CJC Open. 2025 Jul 30;7(10):1314–23. doi: 10.1016/j.cjco.2025.07.013 (PMC12572888; doi:10.1016/j.cjco.2025.07.013)
Supplement: Supplementary Material [file mmc1.pdf]

Supplemental Materials

Supplemental Table S1: All demographic characteristics of survey respondents.

		**UCD**	**UCSF**	**Total**
**Year of training**		N = 44	N = 80	**N = 124**
	Post-graduate year-1	12	23	**35**
	Post-graduate year-2	19	31	**50**
	Post-graduate year-3	12	26	**38**
	Post-graduate year-4	1		**1**
**Age**				
	>32	4	9	**13**
	25-28	13	20	**33**
	29-32	27	51	**78**
**Gender**				
	Female	25	51	**76**
	Male	19	29	**48**
	Other	0	0	**0**
**Training program**				
	Internal Medicine Categorical	30	59	**89**
	Combined Internal Medicine/Psychiatry	4	n/a	**4**
	Internal Medicine Primary Care	10	21	**31**
**Specialty interest**				
	Addiction Medicine	1	7	**8**
	Allergy and Immunology	1		**1**
	Cardiology	5	13	**18**
	Endocrinology, Diabetes, and Metabolism	2		**2**
	Gastroenterology	2	8	**10**
	Hematology and/or Oncology	1	11	**12**
	Hospital Medicine	5	11	**16**
	Infectious Disease	3	1	**4**
	Nephrology	1	1	**2**
	Other	2	2	**4**
	Palliative Care Medicine		1	**1**
	Primary care	7	7	**14**
	Pulmonary and Critical Care Medicine	5	7	**12**

	Rheumatology	2	2	**4**
	Undecided	5	8	**13**
**Location of medical school**				
	Central USA	7	11	**18**
	Northeastern USA	4	24	**28**
	Southern USA	7	10	**17**
	Western USA	26	35	**61**

UCD: University of California, Davis
UCSF: University of California, San Francisco
USA: United States of America

Supplemental Table S2: Participants' choice of beta blocker agent for GDMT initiation classified by institution

Characteristic	Overall	UCD	UCSF	p-value
	N = 124[a]	N = 44[a]	N = 80[a]	
**Beta blocker selected**				0.52[b]
Bisoprolol	1 (0.9%)	1 (2.5%)	0 (0%)	
Carvedilol	27 (25%)	10 (25%)	17 (25%)	
Metoprolol succinate	81 (74%)	29 (73%)	52 (75%)	
Missing	15	4	11	

[a] n (%)

[b] Fisher's exact test

UCD: University of California, Davis
UCSF: University of California, San Francisco

Supplemental Table S3: Participants' choice of beta blocker agent for GDMT initiation classified by region of medical school

**Characteristic**	**Overall**	**Central**	**Northeastern**	**Southern**	**Western**	**p-value**
	N = 124[a]	N = 18[a]	N = 28[a]	N = 17[a]	N = 61[a]	
**Beta blocker selected**						>0.9[b]
Bisoprolol	1 (0.9%)	0 (0%)	0 (0%)	0 (0%)	1 (1.8%)	
Carvedilol	27 (25%)	5 (31%)	4 (18%)	4 (29%)	14 (25%)	
Metoprolol succinate	81 (74%)	11 (69%)	18 (82%)	10 (71%)	42 (74%)	
Missing	15	2	6	3	4	

[a] n (%)

[b] Fisher's exact test

GDMT: Guideline-Direct Medical Therapy

Supplemental Appendix S1:

Thank you for agreeing to participate. In this survey, you will be given 2 short clinical scenarios. Please answer as candidly as possible (your responses are anonymous) and ***answer as if you were the physician responsible for the patient.***

What is your current training year of residency?

- PGY-1
- PGY-2
- PGY-3
- PGY-4
- PGY-5

Age

- <25
- 25-28
- 29-32
- >32

Gender

- Female
- Male
- Non-binary
- Transgender female
- Transgender male
- Other ____

What institution do you currently practice at?

- o UC Davis
- o UC San Francisco

What track in residency are you enrolled in?

- o Internal Medicine Categorical
- o Internal Medicine Primary Care
- o Internal Medicine Combined *(please state which combined program)*

____

Do you have a specialty interest within internal medicine?

- o Addiction Medicine
- o Allergy and Immunology
- o Cardiology
- o Endocrinology, Diabetes, and Metabolism
- o Gastroenterology
- o Geriatric Medicine
- o Hematology and/or Oncology
- o Hospital Medicine
- o Infectious Disease
- o Nephrology
- o Palliative Care Medicine
- o Primary care
- o Pulmonary and Critical Care Medicine
- o Rheumatology
- o Sleep Medicine
- o Sports Medicine
- o *Undecided*
- o *Other* ____

Location of medical school attended

- o Alabama
- o Alaska
- o Arizona
- o Arkansas
- o California
- o Colorado
- o Connecticut
- o Delaware
- o District of Columbia
- o Florida
- o Georgia
- o Hawaii
- o Idaho
- o Illinois
- o Indiana
- o Iowa
- o Kansas
- o Kentucky
- o Louisiana
- o Maine
- o Maryland
- o Massachusetts
- o Michigan
- o Minnesota
- o Mississippi
- o Missouri
- o Montana
- o Nebraska
- o Nevada
- o New Hampshire

o New Jersey

o New Mexico

o New York

o North Carolina

o North Dakota

o Ohio

o Oklahoma

o Oregon

o Pennsylvania

o Puerto Rico

o Rhode Island

o South Carolina

o South Dakota

o Tennessee

o Texas

o Utah

o Vermont

o Virginia

o Washington

o West Virginia

o Wisconsin

o Wyoming

o *Other* ____

Start of Block: PRACTICE 1

CASE 1: You are admitting a 57-year-old woman with heart failure with reduced ejection fraction. Her last ejection fraction on echocardiogram six months ago was 35%. She is complaining of shortness of breath, orthopnea and dyspnea on exertion. She does not have chest pain or lightheadedness. ECG shows no evidence of acute ischemia. - Vitals: BP 98/59 (baseline 118/65), HR 59. She is requiring 4 L O2 via nasal cannula (new oxygen requirement). - Exam: jugular venous distention, pulmonary crackles and 2+ pitting edema. She is 10 pounds above her dry weight. - Labs: BMP is significant for creatinine 1.5 mg/dL (baseline 1.3), serum lactate is unremarkable. Her NT-proBNP is elevated at 1000 pg/ml (no known baseline). You suspect

that she has a heart failure exacerbation. At home she takes metoprolol succinate 50 mg once daily. She is also on empagliflozin, spironolactone and sacubitril-valsartan.

When admitting this patient to the hospital, what would you do with her metoprolol?

- o Hold it on admission and restart before discharge
- o Hold it on admission and restart outpatient
- o Continue it on admission

Display This Question:

If When admitting this patient to the hospital, what would you do with her metoprolol? = Hold it on admission and restart outpatient

Or When admitting this patient to the hospital, what would you do with her metoprolol? = Hold it on admission and restart before discharge

Rank (**by dragging up or down**) the reasons that would lead you to discontinue metoprolol on admission *(with #1 being your top concern)*:
_______ I am concerned that I would be deviating from the practice of my peers (1)
_______ I am concerned that my attending would disagree and request that I stop metoprolol (2)
_______ I am concerned that a consultant would think I made a poor decision (3)
_______ I am concerned about legal implications if an adverse event were to occur (4)
_______ I am concerned about causing cardiogenic shock in my patient (5)
_______ I am concerned that the pharmacist would not dispense the metoprolol prescription (6)
_______ I am concerned that the nurse would not administer the metoprolol (7)
_______ I am concerned that the vital signs are not in ranges where I feel comfortable ordering metoprolol (8)
_______ Other reason (optional): (9)

Display This Question:

If Rank (by dragging up or down) the reasons that would lead you to discontinue metoprolol on admiss... [I am concerned that I would be deviating from the practice of my peers] = 1

"I am concerned that I would be deviating from the practice of my peers" is your top reason for not prescribing metoprolol in this case. Why does deviating from your peers concern you?

____

Display This Question:

If Rank (by dragging up or down) the reasons that would lead you to discontinue metoprolol on admiss... [I am concerned that my attending would disagree and request that I stop metoprolol] = 1

"I am concerned that my attending would disagree and request that I stop metoprolol" is your top reason for not prescribing metoprolol in this case. What are the chances you could change your attending's decision through conversation?

____

Display This Question:

If Rank (by dragging up or down) the reasons that would lead you to discontinue metoprolol on admiss... [I am concerned that a consultant would think I made a poor decision] = 1

"I am concerned that a consultant would think I made a poor decision" is your top reason for not prescribing metoprolol in this case. Why does disagreement by a consultant concern you?

____

Display This Question:

If Rank (by dragging up or down) the reasons that would lead you to discontinue metoprolol on admiss... [I am concerned about legal implications if an adverse event were to occur] = 1

"I am concerned about legal implications if an adverse event were to occur" is your top reason for not prescribing metoprolol in this case. What specific malpractice allegation could you foresee?

____

Display This Question:

If Rank (by dragging up or down) the reasons that would lead you to discontinue metoprolol on admiss... [I am concerned about causing cardiogenic shock in my patient] = 1

"I am concerned about causing cardiogenic shock in my patient" is your top reason for not prescribing metoprolol in this case. What consequences could you foresee?

____

Display This Question:

If Rank (by dragging up or down) the reasons that would lead you to discontinue metoprolol on admiss... [I am concerned that the pharmacist would not dispense the metoprolol prescription] = 1

"I am concerned that the pharmacist would not approve the metoprolol prescription" is your top reason for not prescribing metoprolol in this case. What are the chances you could change the pharmacist's decision through conversation?

____

Display This Question:

If Rank (by dragging up or down) the reasons that would lead you to discontinue metoprolol on admiss... [I am concerned that the nurse would not administer the metoprolol] = 1

"I am concerned that the nurse would not administer the metoprolol" is your top reason for not prescribing metoprolol in this case. What are the chances you could change the nurse's decision through conversation?

____

Display This Question:

If Rank (by dragging up or down) the reasons that would lead you to discontinue metoprolol on admiss... [I am concerned that the vital signs are not in ranges where I feel comfortable ordering metoprolol] = 1

"I am concerned that the vital signs are not in ranges where I feel comfortable ordering metoprolol" is your top reason for not prescribing metoprolol in this case. What consequences do you forsee?

____

Display This Question:

If Rank (by dragging up or down) the reasons that would lead you to discontinue metoprolol on admiss... [Other reason (optional):] = 1

The optional free response you typed is your top reason for not prescribing metoprolol in this case. Why does that dissuade you from ordering metoprolol?

____

In patients with acute heart failure exacerbation, how often do you hold outpatient metoprolol on admission in the absence of new bradycardia, severe hypotension, or signs of shock?

- o Never (0%)
- o Occasionally (1-24%)
- o Often (25-49%)
- o Routinely (50-99%)
- o Always (100%)

In patients with acute heart failure exacerbation, can metoprolol cause cardiogenic shock in the absence of new bradycardia, severe hypotension, or signs of shock?

- o Yes
- o Unsure
- o No

Start of Block: PRACTICE 2

CASE 2: You are admitting a 57-year-old woman complaining of shortness of breath, orthopnea and dyspnea on exertion. She is diagnosed with new heart failure with reduced ejection fraction of 40%. She has jugular venous distention, pulmonary crackles, a new 4L O2 requirement and 2+ pitting edema. Her weight is 10 pounds above her dry weight. Her BMP shows a creatinine of 1.5 mg/dL (baseline 1.2) and the rest of her BMP and CBC are unremarkable. Her high sensitivity troponin is negative. She receives diuresis until she is euvolemic, her symptoms improve, she is off oxygen and labs all normalize. Her BP is 123/70, HR 84, and O2 96% on ambient air. You are planning to start her on guideline-directed medical therapy before discharge.

Which evidence-based beta blocker do you start her on?

- o Metoprolol succinate
- o Carvedilol
- o Bisoprolol

Display This Question:

If Which evidence-based beta blocker do you start her on? = Metoprolol succinate

Choose all the reasons that led you to choose metoprolol:

- ▢ That's what most of my peers have been ordering
- ▢ That's what my attendings typically ask me to order
- ▢ I chose this medication because it's only dosed once a day
- ▢ There is medical evidence to support metoprolol is superior
- ▢ Other reason (optional):

____

Display This Question:

If Which evidence-based beta blocker do you start her on? = Carvedilol

Choose all the reasons that led you to choose carvedilol:

- ▢ That's what most of my peers have been ordering
- ▢ That's what my attendings typically ask me to order
- ▢ I chose this medication because it also offers afterload reduction
- ▢ There is medical evidence to support carvedilol is superior
- ▢ Other reason (optional):

____

Display This Question:

If Which evidence-based beta blocker do you start her on? = Bisoprolol

Choose all the reasons that led you to choose bisoprolol:

- ▢ That's what most of my peers have been ordering
- ▢ That's what my attendings typically ask me to order
- ▢ I chose this medication because it's only dosed once a day
- ▢ There is medical evidence to support bisoprolol is superior
- ▢ Other reason (optional):

____

End of Block: PRACTICE 2

Thank you for taking the time to respond.

If you are interested in entering the drawing for the $25 Amazon gift card, please click here to enter your email:
(Your email will NOT be tied to your survey responses as they are **anonymous**):
The responses from the survey will be stored in Qualtrics. If you choose to enter the drawing for the gift card, you will be directed to another link (above) where you will enter your email in a google survey. The email response in the google survey is separate from the Qualtrics responses and there is no way to link them.
